# Selective Anti-Leishmanial Strathclyde Minor Groove Binders Using an N-Oxide Tail-Group Modification

**DOI:** 10.3390/ijms231911912

**Published:** 2022-10-07

**Authors:** Marina C. Perieteanu, Leah M. C. McGee, Craig D. Shaw, Donna S. MacMillan, Abedawn I. Khalaf, Kirsten Gillingwater, Rebecca Beveridge, Katharine C. Carter, Colin J. Suckling, Fraser J. Scott

**Affiliations:** 1 Department of Pure and Applied Chemistry, University of Strathclyde, Glasgow G1 1XL, UK; 2 Strathclyde Institute of Pharmacy & Biomedical Sciences, University of Strathclyde, Glasgow G4 0RE, UK; 3 Parasite Chemotherapy, Department of Medical Parasitology and Infection Biology, Swiss Tropical and Public Health Institute, 4051 Basel, Switzerland; 4 University of Basel, 4001 Basel, Switzerland

**Keywords:** leishmaniasis, minor groove binders, S-MGB, DNA

## Abstract

The neglected tropical disease leishmaniasis, caused by Leishmania spp., is becoming more problematic due to the emergence of drug-resistant strains. Therefore, new drugs to treat leishmaniasis, with novel mechanisms of action, are urgently required. Strathclyde minor groove binders (S-MGBs) are an emerging class of anti-infective agent that have been shown to have potent activity against various bacteria, viruses, fungi and parasites. Herein, it is shown that S-MGBs have potent activity against *L. donovani*, and that an N-oxide derivation of the tertiary amine tail of typical S-MGBs leads to selective anti-leishmanial activity. Additionally, using S-MGB-219, the N-oxide derivation is shown to retain strong binding to DNA as a 2:1 dimer. These findings support the further study of anti-leishmanial S-MGBs as novel therapeutics.

## 1. Introduction

Leishmaniasis is a parasitic disease found in tropical and subtropical regions, and is transmitted by sandflies of the genus Lutzomyia or Phlebotomus [1,2]. Despite significant advances in public health, leishmaniasis remains a significant problem, and it has been estimated that up to 1.5 M new cases occur each year world-wide [1]. There are three main clinical forms of leishmaniasis: cutaneous leishmaniasis (CL), mucocutaneous leishmaniasis and visceral leishmaniasis (VL, kala-azar), which can be fatal if untreated. In India, *Leishmania donovani* is the only parasite responsible for infections. In VL, the parasite lives in macrophages within the reticuloendothelial system, and is found mainly in the bone marrow, spleen and liver. The parasite can persist in the human host for many years, and following treatment and an apparent absence of symptoms, it can cause dermal lesions resulting in the condition know as post kala-azar dermal leishmaniasis (PKDL). In some cases, PKDL can occur in individuals who have shown no clinical signs of VL, although there is an established elimination programme with the target of reducing the incidence to <1 per 10,000 people in endemic regions [2] which is having some success. However, there are still foci with high endemicity, which could provide reservoirs of drug-resistant parasites that could seed new epidemics. The recent cessation of VL control measures in India caused by the COVID pandemic is predicted to have a major impact on elimination targets [3].

The Indian kala-azar elimination programme and WHO guidelines advise using either amphotericin B or miltefosine for VL treatment. However, these drugs are not ideal; clinical resistance has been reported for both miltefosine and amphotericin B, and miltefosine cannot be administered to pregnant and lactating women and young children. Studies have shown that patients with PKDL can transmit the disease [4], so it is important that active cases are treated effectively as part of an elimination programme. The current recommended treatment for PKDL is sodium stibogluconate (SSG), which is given via an intravenous or intramuscular route for up to 60 days, and is associated with significant toxicity when used for prolonged treatments (>2 months). Alternatively, amphotericin B is given via infusion on alternate days for up 80 doses over 4 months, and is a drug which is also associated with significant side effects [5].

CL is the most abundant form of leishmaniasis and is responsible for one million cases/year. At present, there are no effective treatments for the disease. It can cause life-long scars which cause significant psychological harm [6]. It is clearly important for long-term health and well-being to populate the pipeline of new medicines to treat VL, CL and PKDL. The emergence of strains resistant to even the most recently introduced drugs such as miltefosine is critical, and a lack of investment in this neglected tropical disease means that there are few new drugs being developed [7].

It is essential, therefore, to support a pipeline of anti-leishmanial drugs with novel mechanisms of action to provide treatments where existing medicines and public health measures fail.

**Figure 1:**
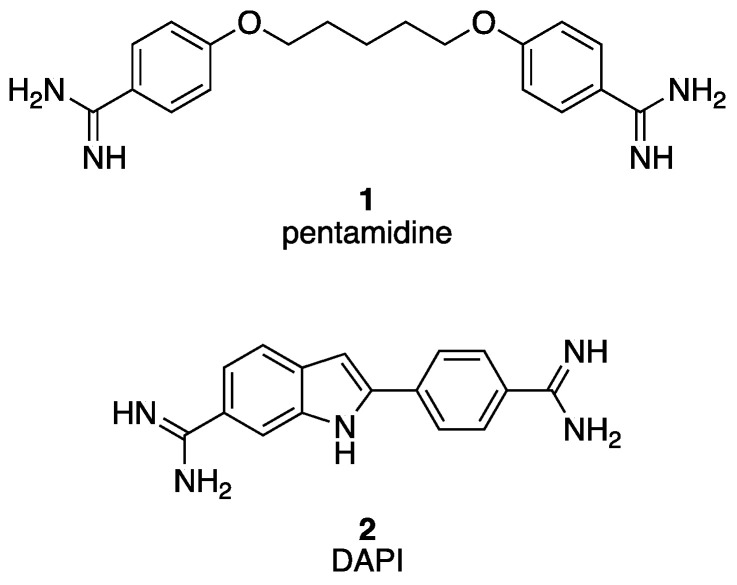
The structure of the anti-parasitic compounds pentamidine **1** and DAPI **2**.

Another drug known to be active against *Leishmania* includes pentamidine (**1**); however, its use is limited by its toxicity and by a growing parasitic resistance to it (Figure 1). Pentamidine (**1**) is of the diamidine classes of drugs, which are known DNA minor groove binders. It has been suggested that its antiparasitic activity be exerted by binding it to DNA and also disrupting the mitochondrial membrane potential [8]. Moreover, work using the fluorescent diamidine, DAPI (**2**), also a minor groove binder, on *Leishmania exicana* demonstrated localisation in the nucleus and kinetoplast DNA [9] (Figure 1).

**Figure 2:**
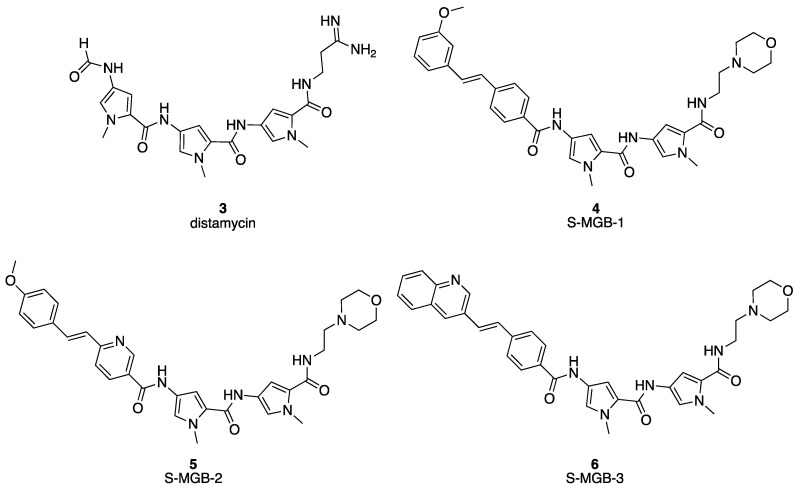
Structures of the natural product distamycin (**3**) and several Strathclyde minor groove binders bearing an isosteric alkene (**4**–**6**).

**Figure 3:**
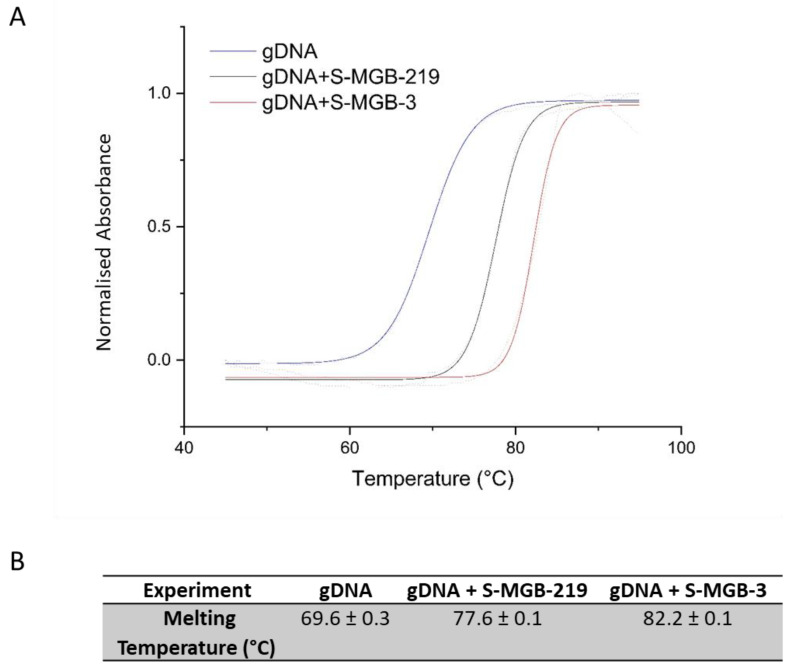
Thermal melt curves of gDNA (salmon), gDNA+S-MGB-3 complex and gDNA+S-MGB-219. (**A**) Exemplar melt curve from one experimental repeat, visually representing the different melt curves of each experiment. Data were fitted with a Boltzmann distribution. (**B**) Melting temperatures of each experiment calculated from fitted Boltzmann distributions using OriginPro 2021. All values are an average of at least *n* = 4 experimental repeats.

Strathclyde minor groove binders (S-MGBs), based loosely upon the structure of the natural product distamycin (**3**), have been shown to have anti-infective activity against bacteria, fungi, parasites and viruses (Figure 2) [10,11,12,13,14,15]. The structural characteristics of the most significant S-MGBs include replacement of the *N*-terminal formyl group in distamycin with an aromatic ring and one of the amide links being replaced with the isosteric alkene, as represented by **4**–**6** (Figure 3). The most developed of these S-MGBs (**6**) has successfully completed a phase IIa clinical trial for the treatment of *Clostridioides difficile* infections [15] (Figure 3). With respect to antiparasitic activity, S-MGBs are effective against many species of trypanosome and are able to cure *Trypanosoma vivax* and *T. congolense* infections in mouse models of the diseases [16]. These results suggest that activity should also be found for *Leishmania spp.*, which is a closely related kinetoplastid genus to *Trypanosoma.* This paper concerns the discovery of a group of anti-leishmanial S-MGBs, derived from potent antibacterial compounds such as **6** via a single, simple structural modification, the formation of an *N*-oxide.

S-MGBs have several highly significant, experimentally proven, advantages. Since the need for new anti-infective agents is caused by the continual emergence of resistant strains of pathogens to established drugs, the exceptionally high resilience of S-MGBs to resistance development is most important. Resilience has been demonstrated in vitro for antibacterial and antitrypanosomal applications and is a consequence of the fact—again, experimentally proven—that S-MGBs target several essential DNA-centric processes in the pathogen [17]. Because S-MGBs have multiple specific molecular targets, the evolution of resistance is greatly repressed. A special property of S-MGBs is that the physicochemical properties can be controlled whilst maintaining essential antiparasitic activity. This, together with their intrinsic resilience to the development of resistance, makes S-MGBs an especially significant compound class in the search for new antileishmanial drugs.

**Scheme 1:**
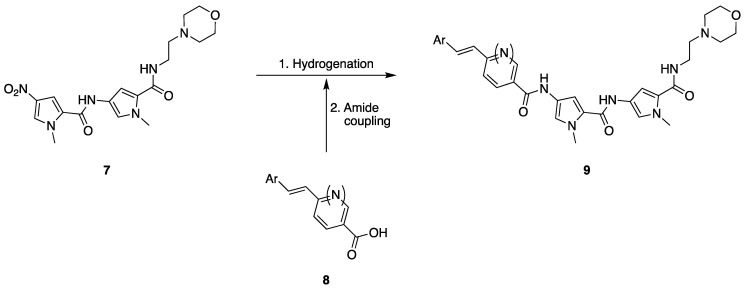
Typical synthesis of S-MGB. Hydrogenation of nitro dimer (**7**) and coupling of resultant amine to carboxylic acid of headgroup (**8**) gives full S-MGB (**9**).

The opportunity was taken to modify standard synthetic approaches for S-MGBs to produce gram quantities of compounds **4**, **5** and **6**. Synthesis of both the alkene-containing head groups and the tail-group dimers can be achieved in gram quantities; however, the challenge for scale-up has always been the final coupling of these two groups. The standard method uses hydrogenation of the nitro group of the tail-group dimer (**7**), followed by HBTU-mediated coupling of **8** and subsequent purification via column chromatography or, more often, HPLC purification (Scheme 1) [18].

## 2. Results and Discussion

### 2.1. Chemistry

**Scheme 2:**
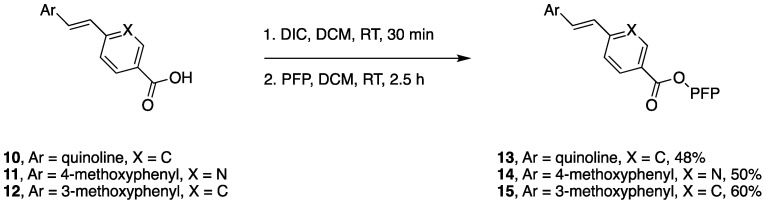
Synthesis of head-group pentafluorophenol esters **13**–**15**.

After a short investigation of coupling methods including using acid chlorides or alternative coupling agents, pentafluorophenyl active esters were selected for coupling. To form the active esters, the appropriate carboxylic acid, either **10**, **11** or **12** (the synthesis of which has been previously reported [18]), was dissolved in anhydrous DCM and the pentafluorophenyl (PFP) ester was formed via coupling with diisopropylcarbodiimide (DIC). A basic work-up to remove excess pentafluorophenol and any unreacted carboxylic acid produced the solid active esters, **13**, **14** and **15** (48–60%) (Scheme 2). These active esters are storable for extended periods of time.

**Scheme 3:**
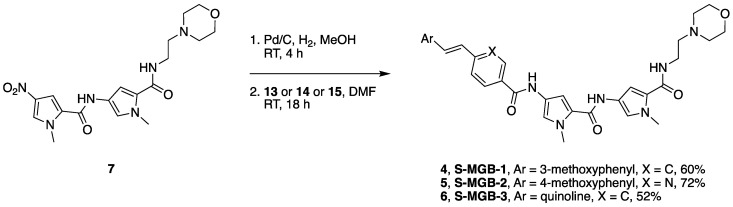
Synthesis of full S-MGBs **1**–**3** and **4**–**6**.

To form the MGBs **S-MGB-1** (**4**), **S-MGB-2** (**5**) and **S-MGB-3** (**6**), the nitro group of the morpholino tail-group dimer (**7**) (the synthesis of which has been previously reported [18]) was reduced to the amine via hydrogenation over Pd/C. Coupling with one equivalent of the appropriate pentafluorophenol ester, either **13, 14** or **15**, gave the S-MGBs (**4, 5** or **6**) in yields of 52–72%, isolated by precipitation and without the need for extensive chromatographic purification (Scheme 3). This is a potentially valuable outcome in the context of scale-up.

**Scheme 4:**
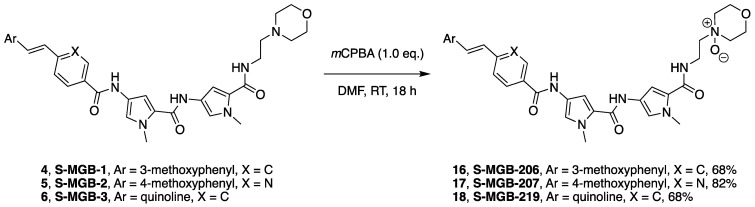
Synthesis of N-oxide tail-group derivatives, **16**–**18**.

Synthesis of the *N*-oxide tail-group-modified S-MGBs was achieved by reacting the appropriate lead compound (**4**, **5** or **6)** with one equivalent of *m*-CPBA in DMF. The N-oxides **S-MGB-206** (**16**), **S-MGB-207** (**17**) and **S-MGB-219** (**18**) were purified by HPLC (Scheme 4).

### 2.2. Biology

All compounds were purified to at least 95% purity by HPLC before the assay. Table 1 shows data for the parent S-MGBs and their *N*-oxides against a relevant group of infectious organisms. The *N*-oxide S-MGBs, interestingly, were not found to be active against *Staphylococcus aureus*, in contrast to their precursors, which include some of the most active S-MGBs against Gram-positive bacteria. In another study in which the structures of **S-MGB-1**, **S-MGB-2** and **S-MGB-3** were truncated by the removal of one pyrrole from the structure, a reduction in Gram-positive activity was also observed [19]. In other studies, antifungal activity was not found for the *N*-oxides either (data not reported).

The human voltage-sensitive K+ channel hERG is involved in cardiac action potential repolarisation, controlling the QT interval of the electrocardiogram. The lack of significant inhibition of hERG is an important property to consider in progressing a drug candidate, in order to avoid drug-related arrhythmia. The table also shows the effect of the six S-MGBs on the hERG ion channel, measured using the percentage displacement of [^3^H]astemizole by the S-MGBs at 10 μM. Data for **S-MGB-1**, **S-MGB-2** and **S-MGB-3** have already been published, but are included in the table for comparison [20]. These three S-MGBs contain *t*-amino tail groups and the low but measurable activity was not surprising. The neutral *N*-oxide tail group could be expected to lend the new S-MGBs lower activity at the hERG ion channel, and this is what was found: **S-MGB-206**, **S-MGB-207** and **S-MGB-219** show between 13 and 21% lower inhibition of astemizole binding than the parent compounds **S-MGB-1**, **S-MGB-2** and **S-MGB-3**.

The anti-infective activity of the *N*-oxides, in particular, their lack of activity against bacteria and fungi, suggests that they might be of interest as selective antiparasitic compounds. The previously mentioned truncated S-MGBs were also found to be selective antiparasitic compounds, and thus, both truncation and *N*-oxide strategies are comparable in this regard. The differences in activity between the *N*-oxides and *t*-amino S-MGBs also raise the question of whether *N*-oxide-containing S-MGBs bind to DNA in a similar manner to their *t*-amino precursors. It is therefore pertinent to investigate binding to DNA using biophysical methods.

### 2.3. Thermal Melting Study

As DNA is the biological target of S-MGBs, it is necessary to demonstrate target engagement for the newly synthesised compounds, and their comparators. The *N*-oxides and their precursor tertiary amines were evaluated for their relative DNA binding affinities to the AT-rich self-complementary oligomer d(5′-CGCATATATGCG-3′) by measuring their effects on its melting temperature (Table 2).

All three *N*-oxides showed a measurable increase in T_m_ consistent with binding to the AT-rich oligomer selected. The strong binding by this measure of **S-MGB-219** is notable and can be associated with the quinolyl head group; this head group has been frequently found to support strong DNA binding in S-MGBs [21]. The test oligonucleotide, d(5′-CGCATATATGCG-3′), is designed to identify binders to AT-rich regions; however, to expand the scope of the thermal binding assay, **S-MGB-3** and **S-MGB-219** were also investigated using genomic DNA from salmon, using **S-MGB-3** as a comparator (Figure 3). Salmon is commonly used as an easily accessible, cheap source of genomic DNA, and has been used in early-stage target-binding studies of S-MGBs previously [11]. **S-MGB-219** had a substantial T_m_ increase of 8.0 °C, compared to 12.6 °C for **S-MGB-3**, the latter of which is in line with previous work on **S-MGB-3** (manuscript in preparation). These results suggest that both **S-MGB-219** and **S-MGB-3** are strong DNA binders. However, the trend of lower binding strength of the N-oxide compared to the parent compound was observed in both experiments (genomic DNA and short oligo) for **S-MGB-219** compared to **S-MGB-3**.

### 2.4. Native Mass Spectrometry

**Figure 4:**
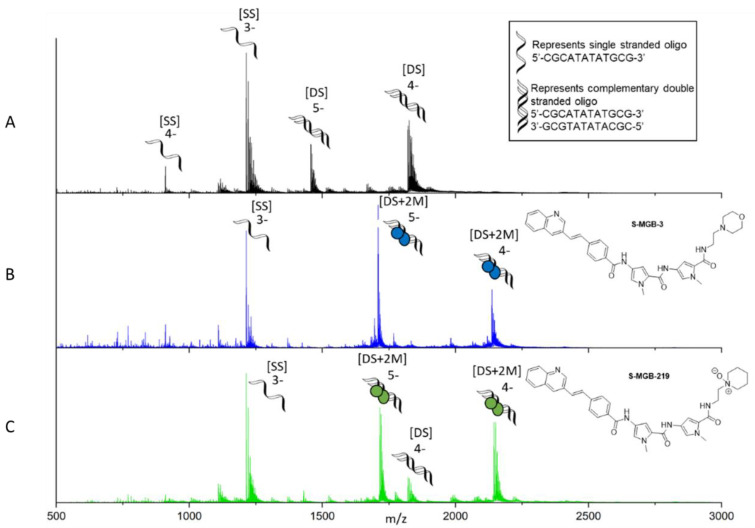
Characterisation of S-MGB-BP3 and S-MGB-219 binding to double-stranded DNA as a dimer via nMS. Spectra of DNA sequence 5′-CGCATATATGCG-3′ (9 μM DNA, 100 μM KCl and 1% DMSO) sprayed from ammonium acetate (150 mM, pH 7) in the (**A**) absence and (**B**) presence of 100 μM S-MGB-BP-3, or (**C**) of 100 μM S-MGB-219. (**A**) Single-stranded DNA (denoted by [SS]) is present in charge states 4- and 3-, and double-stranded DNA (denoted by [DS]) is present in charge states 5- and 4-. (**B**) [SS] is present in charge states 3- and 4-. Each [DS] molecule is seen to bind 2xS-MGB molecules (denoted by [DS+2M]) and is present in charge states 5-and 4-. (**C**) [SS] is present in charge states 3- and 4-. Each [DS] molecule is seen to bind 2xS-MGB molecules (denoted by [DS+2M]) and is present in charge states 5-and 4-. Double-stranded DNA (denoted by [DS]) is present in charge state 4-.

To complement the melting temperature measurements and to further probe the nature of the binding of **S-MGB-219** to DNA, native mass spectrometry (nMS) experiments were carried out. A particular property of S-MGBs’ binding to DNA is their self-association as antiparallel head-to-tail dimers [22]. nMS is an informative method with which to investigate the stoichiometry of MGBs binding to a short DNA oligomer and has previously been used to confirm the binding of **S-MGB-3** as a dimer (manuscript in preparation). Therefore, nMS was used to compare the binding of **S-MGB-219** to **S-MGB-3**. Using the same evaluation oligomer as for the thermal melting experiments, d(5′-CGCATATATGCG-3′), an nMS spectrum was obtained for **S-MGB-219**, and compared to that of **S-MGB-3** (Figure 4, Supplementary Materials Tables S1 and S2).

The DNA oligomer itself, 5′-CGCATATATGCG-3′, was shown by nMS to have both single-stranded DNA [SS] in charge states 4- and 3-, and double-stranded DNA [DS] in charge states 5- and 4- (Figure 4), which is typical of these experiments [11]. The nMS of DNA sequence 5′-CGCATATATGCG-3′ in the presence of **S-MGB-219** showed that it bound to double-stranded DNA as a dimer [DS+2M] in charge states 5- and 4- (Figure 4). As previously mentioned, we have reported a similar nMS experiment of S-MGB-3 in a separate study (manuscript in preparation) and the similarities between the observations for **S-MGB-219** and those for **S-MGB-3** are notable. The results indicate that both S-MGBs selectively bind to double-stranded DNA as a dimer only. Perhaps in line with the lower strength of binding observed for **S-MGB-219** compared to **S-MGB-3** in the thermal melt experiments, there is evidence of a small amount of unbound double-stranded DNA [DS] for **S-MGB-219** (Figure 4). Despite this, no evidence was found for **S-MGB-219** bound to double-stranded DNA as a monomer, [DS+1M].

## 3. Conclusions

The biological activity and biophysical measurements described above provide data that lead to several significant points. Firstly, *N*-oxide is a competent tail group for biologically active minor groove binders of the S-MGB type. The anti-infective activity is comparable with that of the corresponding *t*-amino tail groups in the anti-parasitic context. On the other hand, antibacterial and antifungal activity are absent. This leads to the possibility that *N*-oxide-bearing S-MGBs might be of interest as selective anti-parasitic compounds. Since *N*-oxide is a probable phase 1 metabolite of the *t*-amine, it is also possible that such metabolites might prolong anti-infective activity *in vivo* for susceptible pathogens. Secondly, the biophysical measurements show that the DNA-binding properties of an *N*-oxide bearing S-MGB, **S-MGB-219** are very similar to those of the parent *t*-amine, **S-MGB-3**. This suggests that the *N*-oxides could be acting via a similar mechanism of action to that of the *t*-amines. A positively charged tail group is therefore not essential for DNA binding and anti-infective activity, a conclusion that has been drawn before for S-MGBs with neutral but highly polar tail groups [20]. Further studies of both anti-leishmanial S-MGBs and of the properties of *N*-oxides in S-MGBs will be supported by the results in this paper. In particular, it will be necessary to fully investigate the cytotoxicity profiles of N-oxide S-MGBs in comparison to their tertiary amine comparators to ensure that selectivity between parasite and mammalian cells can be achieved. Additionally, a more sophisticated assay sequence taking account of intracellular infection and parasite life cycles will be used in future studies.

## 4. Experimental Procedure

### 4.1. Biological Evaluation

#### 4.1.1. Antibacterial Assay

The minimum inhibitory concentration (MIC) against *S. aureus* ATCC 43300 and *E. faecalis* ATCC 51299 was measured by transferring a two-fold serial dilution of the samples into 96-well non-binding surface plate (Corning #3640). The bacteria were cultured in cation-adjusted Mueller Hinton broth (CAMHB) overnight at 37 °C, diluted 40-fold and incubated for a further 1.5–3 h at 37 °C. The resultant mid-log phase cultures were diluted and added to each well of the compound-containing plates, giving a cell density of 5 × 10^5^ CFU mL^−1^, measured via absorbance at 600 nm (OD600), and a final compound concentration range of 50–0.0195 μM. All the plates were covered and incubated at 37 °C for 18 h without shaking. The inhibition of bacterial growth was determined by OD600, using a Tecan Infinite M Nano plate reader. The percentage of growth inhibition was calculated for each well, using the negative control (medium only) and positive control (bacteria without inhibitors) on the same plate. The MIC was determined as the lowest concentration at which growth was fully inhibited, defined by an inhibition of 80%. Each MIC determination was carried out in triplicate, on separate days.

#### 4.1.2. Anti-Trypanosoma Assay

The bloodstream form *T. b. brucei* (Lister 427) was cultured in HMI-11 medium (Gibco) supplemented with 10% heat-inactivated FBS (Gibco), at 37 °C in a humidified 5% CO_2_ environment.

The EC_50_ values were determined using the in vitro alamarBlue assay. *T. b. brucei* parasites (2 × 10^4^ cells per mL), were seeded into serial dilutions of the test compounds to a final volume of 200 μL and incubated for 48 h, after which 20 μL of 0.49 mM resazurin dye (Sigma-Aldrich, St. Louis, MO, USA) was added and the cells were incubated for a further 24 h. The reduction of resazurin was measured using a fluorimeter (FLUOstar Optima, BMG Labtech, Ortenberg, Germany) at 544 nm excitation and 590 nm emission wavelengths. The EC50 values were determined using Prism 5 software (GraphPad, San Diego, CA, USA). All experiments were carried out on at least three independent occasions.

#### 4.1.3. Anti-Leishmania Assay

The compounds were screened in vitro for their antileishmanial activity against the intracellular amastigote stage [23,24]. Briefly, bone marrow-derived macrophages (0.5 × 10^5^ cells per well) were infected using a 20:1 host cell: parasite ratio with luciferase-expressing *L. donovani* LV82 luc1 (MHOM/ET/67:LV82). The medium was changed at 24 h post-infection to remove free parasites, and infected cells were treated with medium alone (controls, *n* = 6) or doubling dilutions of DMSO (*n* = 3, starting at 2% or 2.5% *v*/*v*) or MGB compounds (*n* = 3 or 6, starting at 25 or 20 μg mL^−1^, prepared from a 1 mg mL^−1^ DMSO stock solution). Amphotericin B solution was used as a positive control and was used at a starting concentration of 1 μM. The medium was removed from each sample at 72 h post-infection and replaced with 150 μL luciferin solution (150 μg mL^−1^ luciferin in serum-free medium). The amount of bioluminescent signal emitted/well was determined and used to calculate the mean suppression in the bioluminescent signal for each test sample compared to the mean control values. The EC_50_ values were calculated and the mean EC_50_ was calculated from the three separate experiments [25].

### 4.2. hERG Evaluation

#### 4.2.1. Preparation of Cells

hERG-transfected HEK293 cells were kindly gifted by Dr. J. Mitcheson, University of Leicester. The cell cultures were maintained in Gibco Dulbecco’s Modified Eagle Medium (D-MEM) + GlutaMAX1^TM^ medium supplemented with 10% foetal bovine serum (BSA), 1% penicillin–streptomycin and 0.8% Geneticin in a Sanyo humidified incubator (37 °C, 5% CO_2_). Once at 80–90% confluency, the cells were lifted using a PBS-based, enzyme-free Cell Dissociation Buffer. The cell suspension was counted using a Bright Line Haemocytometer and centrifuged at 1000 rpm for 5 min, and the supernatant discarded. The pellet was resuspended in ice-cold Tris-HCl, pH 7.4 (20 mM), with 1% phenylmethylsulfonyl fluoride (PMSF). The cells were homogenised using a Brinkman Polytron and the homogenate centrifuged using a Beckman Coulter Optima L-70K Ultracentrifuge or an L-100 XP Ultracentrifuge at 200,000*g* × for 40 min at 4 °C. Then, they were resuspended in buffer (10 mM HEPES, pH 7.4, 130 mM NaCl, 5 mM KCl, 0.8 mM MgCl_2_, 10 mM glucose, 0.1% BSA) and the aliquots quick-frozen in liquid N_2_ and stored at 80 °C.

#### 4.2.2. hERG Cell-Binding Assay

[^3^H]-Astemizole binding in hERG-transfected HEK293 cells was performed in a 300 mL total volume according to the following conditions: cells (~200,000 cells per assay tube, 100 mL), [^3^H]- astemizole (0.5 nM, 100 mL), incubation buffer (10 mM HEPES, pH 7.4, 130 mM NaCl, 5 mM KCl, 0.8 mM MgCl_2_, 10 mM glucose, 0.1% BSA, 100 mL) for the total binding or drug compound (10 mM, 100 mL) for the single-point assay. Non-specific binding was determined using nonradiolabelled astemizole (10 mM, 100 mL). The tubes were incubated for 1 h at 30 °C, after which time the tube contents were rapidly filtered using a Brandel Cell Harvester under vacuum, then through GF/B filters, and washed with ice-cold incubation buffer (2 mL × 5, see above). The radioactivity retained on the filters (pre-soaked in 0.5% PEI) was measured via counting in scintillation fluid (4 mL, Tri-Carb 1500 Packard; Emulsifier-Safe).

### 4.3. UV-Vis DNA Thermal Melting Experiments

#### 4.3.1. Short DNA Oligo

The DNA oligonucleotide sequence 5′-CGCATATATGCG-3′ was purchased in lyophilised form from Alpha DNA, Canada, and used without further purification, with purity assessed via NMR. The DNA oligomer was melted at a rate of 0.5 °C/min in 10 mM PBS buffer solution (pH 7.4) with 50 mM NaCl on a Cary 300 BIO UV–visible spectrophotometer fitted with a Peltier temperature controller. The programs were set and the data were processed using Cary WinUV software. The duplex oligomer was made to a concentration of 6 × 10^−6^ M, heated to 90 °C and allowed to cool to room temperature unaided. This was mixed with sufficient S-MGB to give the appropriate ratio of 2 MGB: 1 DNA. For the reference melting temperature, no S-MGB was added. Samples were heated from 10 °C to 90 °C and cooled from 90 °C to 10 °C, with the spectra being recorded at 260 nm during both of these cycles. The melting temperatures (Tm) of the duplex oligomers were determined by fitting a sigmoidal function using a Boltzmann distribution in OriginPro. This process was repeated a total of three times, which showed an average error no worse than ±0.5 °C

#### 4.3.2. Genomic DNA

Salmon DNA (D1626, Sigma-Aldrich) at 1 mg/mL in 1 mM phosphate buffer (pH 7.4) containing 0.27 mM KCl and 13.7 mM NaCl (P4417, Sigma-Aldrich) was annealed at 90 °C for 10 min. MGB-BP-3 at 10 mM in DMSO was diluted with the same phosphate buffer, and combined with the salmon DNA stock, to yield a single sample with 10 µM S-MGB and 0.02 mg/mL gDNA in 1 mM phosphate buffer containing 0.27 mM KCl and 13.7 mM NaCl. Control samples containing only MGB-BP-3 or gDNA were prepared, respectively. The samples were melted at a rate of 0.5 °C/min from 45 °C to 90 °C, with spectra recorded at 260 nm on a UV-1900 UV-vis spectrophotometer fitted with a Peltier temperature controller (Shimadzhu, Kyoto, Japan), using LabSolutions (Tm Analysis) software. The melting temperatures (Tms) of the MGB-BP-3:DNA complexes were determined by fitting a sigmoidal function using a Boltzmann distribution in OriginPro. Two independent experiments were carried out and the values quoted with an error no worse than ±0.5 °C.

### 4.4. Native Mass Spectrometry Experiments

DNA sample preparation: The DNA oligonucleotide sequence 5′-CGCATATATGCG-3′ was purchased in lyophilised form from Alpha DNA, Montreal, Canada, and used without further purification; its purity was assessed via NMR. We prepared 100 μM stock solutions of DNA with 150 mM ammonium acetate buffer solution (Fisher Scientific, Loughborough, Leicestershire, UK) and 2 mM potassium chloride solution (Fisher Scientific, Loughborough, Leicestershire, UK). This solution was annealed at 90 degrees for 10 min and allowed to cool to room temperature. A total of 10 mM S-MGB stock in 100% DMSO (Sigma-Aldrich, St. Louis, MO, USA) was diluted to a 1 mM S-MGB solution with 150 mM ammonium acetate. The final samples were prepared from this solution to yield final concentrations of 9 μM DNA, 100 μM KCl, 100 μM S-MGB and 1% DMSO. DNA solutions containing no S-MGB included 1% DMSO and were used as controls.

Mass spectrometry measurements: Native mass spectrometry experiments were carried out using a Synapt G2Si instrument (Waters, Manchester, UK) with a nanoelectrospray ionisation source (nESI). Mass calibration was performed using a separate infusion of NaI cluster ions. The solutions were ionised from a thin-walled borosilicate glass capillary (i.d. 0.78 mm, o.d. 1.0 mm) (Sutter Instrument Co., Novato, CA, USA) pulled in-house to an nESI tip with a Flaming/Brown micropipette puller (Sutter Instrument Co., Novato, CA, USA). A negative potential in range of 1.0–1.2 kV was applied to the solution via a thin platinum wire (diameter, 0.125 mm; Goodfellow, Huntingdon, UK). The following instrument parameters were used for the DNA: S-MGB-219 complex: capillary voltage, 1.1 kV; sample cone voltage, 110 V; source offset, 130 V; source temperature, 40 °C; trap collision energy, 5.0 (V); and trap gas, 4.0 mL/min. For DNA with no MGB present, a capillary voltage of 1.0 kV was applied to the sample. A sample cone voltage of 80 V, source offset of 95 V, source temperature of 40 °C, trap collision energy of 3.0 (V) and trap gas at 4.0 mL/min were used. The data were processed using Masslynx V4.2 and OriginPro 2021, and the figures were produced using chemdraw.

### 4.5. Chemistry

#### 4.5.1. General Experimental Methods

1H and 13C NMR spectra were measured on a Bruker DPX-500 MHz spectrometer with chemical shifts given in ppm (d values), relative to proton and carbon traces in solvent. Coupling constants are reported in Hz. The data are presented as follows: chemical shift, multiplicity (s = singlet, d = doublet, t = triplet, q = quartet, m = multiplet, br = broad, app = apparent), coupling constant (s) in Hertz (Hz) and integration. Chemical shifts (*δ*) were recorded relative to the residual DMSO-*d*_6_ (*δ* = 2.50 in ^1^H NMR and *δ* = 35.2 in ^13^C NMR). The IR spectra were recorded using a PerkinElmer 1 FT-IR spectrometer. The Mass spectra were obtained using a Jeol JMS AX505. Anhydrous solvents were obtained from a Puresolv purification system, from Innovative Technologies, or purchased as such from Aldrich. Melting points were recorded on a Reichert hot-stage microscope, and were uncorrected. Chromatography was carried out using 200–400 mesh silica gels, or using reverse-phase HPLC on a water system using a C18 Luna column; the gradients are given in individual entries below. The purities of all the new truncated MGBs were more than 96%, which was confirmed by HPLC.

#### 4.5.2. General Pentafluorophenol Ester Synthesis

The carboxylic acid of the head group (**10**, **11** or **12**) (10.14 mmol) was dissolved in DCM (60 mL, dry) to which we added DIC (1.920 g, 15.21 mmol), and this was left to stir for 30 min. Pentafluorophenol (1.866 g, 10.14 mmol) was then added slowly and the reaction monitored via TLC using 1:1; ethylacetate: hexane. After about 2 h, the reaction mixture was washed with saturated aqueous potassium carbonate solution (3 × 10 mL) and the organic phase subjected to rotary evaporation to yield an off-white solid. This was washed with ether to yield the desired material in pure form.

2,3,4,5,6-pentafluorophenyl 4-[(E)-2-(3-quinolinyl)ethenyl]benzoate (**13**)

Off-white solid; yield: 48%; m.p.: 102–105 °C; n_max_ (KBr, cm^−1^): 3461, 3414, 1741, 1603, 1594, 1518, 1247, 1053; δ_H_ (CDCl_3_, 500 MHz) δ: 9.18 (1H, d, J = 2.1), 8.2–8.3 (3H, m), 8.13 (1H, d, J = 8.5), 7.87 (1H, d, J = 8.5), 7.74 (3H, d, J = 8.5), 7.60 (1H, t, J = 8.5), 7.43 (2H, m). HRMS (FAB): found 441.0786; calcd. for C_24_H_13_NO_3_F_5_ [M]^+^: 441.0788.

2,3,4,5,6-pentafluorophenyl 6-[(E)-2-(4-methoxyphenyl)ethenyl]nicotinate (**14**)

Off-white solid; yield: 50%; m.p.: 152–154 °C; n_max_ (KBr, cm^−1^): 3328, 3031, 2930, 2851, 1756, 1704, 1587, 1519, 1257, 1176, 1059, 991; δ_H_ (DMSO, 500 MHz) δ: 9.33 (1H, d, J = 2.0), 8.37 (1H, dd, J = 8.0, 2.0), 7.84 (1H, d, J = 16.0), 7.59 (1H, d, J = 8.0), 7.49 (2H, d, J = 9.0), 7.13 (1H, d, J = 16.0), 6.95 (2H, d, J = 9.0), 3.87 (3H, s); ^13^C NMR (DMSO, 125 MHz) δ: 161.5, 161.3, 160.7, 151.9, 142.1 (m), 140.0 (m), 139.1(m), 138.5, 137.0 (m), 136.7, 129.1, 128.6, 124.3, 121.3, 120.3, 114.3, 55.3. HRMS (FAB): found 421.0739; calcd. for C_21_H_13_NO_3_F_5_ [M]^+^: 421.0737.

2,3,4,5,6-pentafluorophenyl 4-[(E)-2-(3-methoxyphenyl)ethenyl]benzoate (**15**)

Off-white solid; yield: 60%; m.p.: 110–122 °C; n_max_ (KBr, cm^−1^): 2962, 1755, 1604, 1578, 1520, 1284, 1258, 1227, 1049; δ_H_ (DMSO, 500 MHz) δ: 8.18 (2H, d, J = 8.4), 7.66 (2H, d, J = 8.4), 7.21–7.42 (3H, m), 7.12–7.19 (2H, m), 7.11 (1H, m), 6.90 (1H, dd, J = 7.8, J = 2.0), 3.88 (3H, s); ^13^C NMR (DMSO, 125 MHz) δ: 162.3, 160.0, 143.6, 142.2 (m), 140.5 (m), 138.9 (m), 137.8, 136.8 (m), 132.4, 131.2, 129.8, 127.3, 126.7, 125.4, 119.6, 114.2, 112.1, 55.3. HRMS (FAB): found 420.0784; calcd. for C_22_H_14_NO_3_F_5_ [M]^+^: 420.0785.

#### 4.5.3. General MGB Synthesis

4-({4-[(E)-2-(3-Methoxyphenyl)ethenyl]benzoyl}amino)-1-methyl-N-[1-methyl-5-({[2-(4-morpholinyl)ethyl]amino}carbonyl)-1H-pyrrol-3-yl]-1H-pyrrole-2-carboxamide^90^ **S-MGB-1 (4)**

1-Methyl-*N*-[1-methyl-5-({[3-(4-morpholinyl)propyl]amino}carbonyl)-1*H*-pyrrol-3-yl]-4-nitro-1*H*-pyrrole-2-carboxamide (**7**) (160 mg, 0.396 mmol) was dissolved in methanol (35 mL) at 0 °C under nitrogen. Pd/C-10% (110 mg) was added portionwise with stirring under nitrogen at 0 °C. The reaction mixture was hydrogenated for 3 h at room temperature and under atmospheric pressure. The catalyst was removed over Kieselguhr and the solvent was removed under reduced pressure to give the amine. This was dissolved in DMF (2 mL, dry) to which we added 2,3,4,5,6-pentafluorophenyl 4-[€-2-(3-methoxyphenyl)ethenyl]benzoate (**15**) (166 mg, 0.396 mmol). The reaction mixture was heated to 50 °C for 2 h; then, the reaction mixture was left to be stirred at room temperature overnight. DMF was removed under reduced pressure and the crude product was treated with ethyl acetate and saturated aqueous solution of potassium carbonate. The precipitated solid was collected, washed with distilled water and dried to give the required product as a pale-yellow solid (145 mg, 60%). The data obtained for this compound are consistent with previous preparations.

M.p. >230 °C. n_max_ (KBr, cm^−1^): 1681, 1642, 1577, 1464, 1435, 1404, 1266, 1202, 1134. δ_H_ (DMSO-d_6_): 10.32 (1H, s), 9.97 (1H, s), 9.68 (1H, br), 8.23 (1H, t, *J* = 5.6), 7.97 (2H, d, *J* = 8.4), 7.75 (2H, d, *J =* 8.4), 7.3–7.29 (4H, m), 7.22 (2H, m), 7.12 (1H, d, *J =* 1.5), 7.00 (1H, d, *J =* 1.5), 6.90 (1H, dd, *J =* 3.7, *J =* 1.5), 3.99 (2H, m), 3.88 (3H, s), 3.83 (3H, s), 3.81 (3H, s), 3.67–3.55 (6H, m), 3.27 (2H, m), 3.14 (2H, m). HRMS (FAB): found 611.2971; calculated for C_34_H_40_N_6_O_5_^+^ (M + H): 611.2982.

6-[(E)-2-(4-Methoxyphenyl)ethenyl]-N-[1-methyl-5-({[1-methyl-5-({[2-(4-morpholinyl)ethyl]amino}carbonyl)-1H-pyrrol-3-yl]amino}carbonyl)-1H-pyrrol-3-yl]nicotinamide^90^ **S-MGB-2 (5)**

1-Methyl-*N*-[1-methyl-5-({[3-(4-morpholinyl)propyl]amino}carbonyl)-1*H*-pyrrol-3-yl]-4-nitro-1*H*-pyrrole-2-carboxamide **7** (200 mg, 0.495 mmol) was dissolved in methanol (35 mL) at 0 °C under nitrogen. Pd/C-10% (100 mg) was added portionwise with stirring under nitrogen at 0 °C. The reaction mixture was hydrogenated for 4 h at room temperature and atmospheric pressure. The catalyst was removed over Kieselguhr and the solvent was removed under reduced pressure to give the amine. This was dissolved in DMF (2 mL, dry) to which we added 2,3,4,5,6-pentafluorophenyl 6-[(*E*)-2-(4-methoxyphenyl)ethenyl]nicotinate (**14**) (140 mg, 0.332 mmol). The reaction mixture was heated to 50 °C for 2 h; then, the reaction mixture was left to be stirred at room temperature overnight. DMF was remover under reduced pressure and the crude product was treated with ethyl acetate and saturated solution of potassium carbonate. The precipitated pale-yellow solid was collected, washed with distilled water and dried to give the required product as a pale-yellow solid (146 mg, 72%). The data obtained for this compound are consistent with previous preparations.

M.p. >230 °C. n_max_ (KBr, cm^−1^): 3427, 1673, 1588, 1402, 1253, 1202, 1174, 832, 720. δ_H_ (DMSO-d_6_): 10.48 (1H, s), 9.98 (2H, s & br), 9.07 (1H, s), 8.28 (1H, d, *J =* 2.3), 8.26 (1H, d, *J =* 2.3), 7.77 (1H, d, *J =* 16.0), 7.67 (3H, d, *J =* 8.8), 7.35 (1H, d, *J =* 1.6), 7.27 (1H, d, *J =* 16.0), 7.22 (1H, d, *J =* 1.6), 7.12 (1H, d, *J =* 1.6), 7.01 (3H, m), 4.01 (2H, m), 3.88 (3H, s), 3.83 (3H, s), 3.80 (3H, s), 3.73 (2H, m), 3.56 (4H, m), 3.27 (2H, m), 2.99 (2H, m). HRMS (FAB): found 612.2990; calculated for C_34_H_39_O_5_N_6_^+^ (M + H): 612.2982.

1-methyl-N-[1-methyl-5-({[2-(4-morpholinyl)ethyl]amino}carbonyl)-1H-pyrrol-3-yl]-4-({4-[(E)-2-(3-quinolinyl)ethenyl]benzoyl}amino)-1H-pyrrole-2-carboxamide^90^ **S-MGB-3 (6)**

1-Methyl-*N*-[1-methyl-5-({[3-(4-morpholinyl)propyl]amino}carbonyl)-1*H*-pyrrol-3-yl]-4-nitro-1*H*-pyrrole-2-carboxamide **7** (0.300 g, 0.742 mmol) was dissolved in methanol (50 mL and DMF (10 mL) at 0 °C under nitrogen. Pd/C-10% (178 mg) was added portionwise with stirring under nitrogen at 0 °C. The reaction mixture was hydrogenated for 4 h at room temperature and atmospheric pressure. The catalyst was removed over Kieselguhr and the solvent was removed under reduced pressure to give the amine. To the DMF solution, 2,3,4,5,6-pentafluorophenyl 4-[(*E*)-2-(3 quinolinyl)ethenyl] benzoate **13** (328 mg, 0.742 mmol) was added. The reaction mixture was heated to 50 °C for 2 h; then, the reaction mixture was left to be stirred at room temperature overnight. The DMF was removed under reduced pressure and the crude product was treated with ethyl acetate containing 5% methanol and saturated solution of potassium carbonate. The precipitated solid was collected, washed with distilled water and dried to give the required product as a pale-yellow solid (244 mg, 52%). The data obtained for this compound are consistent with previous preparations.

M.p. >230 °C. n_max_ (KBr, cm^−1^): 1681, 1642, 1577, 1464, 1435, 1404, 1266, 1202, 1134. δ_H_ (DMSO-d_6_): 10.35 (1H, s), 9.98 (1H, s), 9.55 (1H, br), 9.28 (1H, d, *J =* 2.0), 8.59 (1H, d, *J =* 2.0), 8.23 (1H, t, *J =* 8.0), 8.05–7.97 (5H, m), 7.83–7.75 (4H, m), 7.70–7.60 (4H, m), 7.34 (1H, d, *J =* 1.7), 7.21 (1H, d, *J =* 1.7), 7.13 (1H, d, *J =* 1.7), 7.01 (1H, d, *J =* 1.7), 4.03–3.99 (2H, m), 3.88 (3H, s), 3.83 (3H, s), 3.69–3.63 (2H, m), 3.59–3.54 (4H, m), 3.28 (2H, m), 3.15 (2H, m). HRMS (FAB): found 632.2982; calculated for C_36_H_38_N_7_O_4_^+^ (M + H): 632.2985.

#### 4.5.4. General MGB Tail-Group Modification

HPLC Procedure for N-Oxides (Table 3).

4-({4-[(E)-2-(3-methoxyphenyl)ethenyl]benzoyl}amino)-1-methyl-N-[1-methyl-5-({[2-(4-oxido-4-morpholinyl)ethyl]amino}carbonyl)-1H-pyrrol-3-yl]-1H-pyrrole-2-carboxamide **S-MGB-206 (18)**.

4-({4-[(*E*)-2-(3-Methoxyphenyl)ethenyl]benzoyl}amino)-1-methyl-*N*-[1-methyl-5-({[2-(4-morpholinyl)ethyl]amino}carbonyl)-1*H*-pyrrol-3-yl]-1*H*-pyrrole-2-carboxamide, **S-MGB-1 (4)**, (10 mg, 16.4 mmol) was dissolved in DMF (1 mL, anhydrous) to which we added *m*-CPBA (2.8 mg, 16.4 mmol). This was left to stir overnight after which it was purified directly by HPLC to obtain the desired product (7 mg, 68%). Purity after HPLC: >95%. m.p. >230 °C. n_max_ (KBr, cm^−1^): 3279, 2954, 2867, 1672, 1642, 1632, 1526, 1433, 1399, 1265, 1131. δ_H_ (DMSO-d_6_): 12.41 (1H, s), 10.32 (1H, s), 9.97 (1H, s), 8.31 (1H, t, *J* = 5.8), 7.96 (2H, d, *J* = 8.4), 7.73 (2H, d, *J =* 8.4), 7.3–7.4 (4H, m), 7.20–7.25 (3H, m), 7.11 (1H, d, *J =* 1.5), 6.97 (1H, d, *J =* 1.5), 6.87 (1H, d, *J =* 1.5), 3.95–3.98 (2H, m), 3.87 (3H, s), 3.81–3.85 (2H, m), 3.83 (3H, s), 3.81 (3H, s), 3.65–3.72 (4H, m), 3.28 (2H, m), 3.15 (2H, m). HRMS (FAB): found 627.2916; calculated for C_34_H_39_O_6_N_6_^+^ (M + H): 627.2926.

##### [(E)-2-(4-methoxyphenyl)ethenyl]-5-({[1-methyl-5-({[1-methyl-5-({[2-(4-oxido-4-morpholinyl)ethyl]amino}carbonyl)-1H-pyrrol-3-yl]amino}carbonyl)-1H-pyrrol-3-yl]amino}carbonyl)pyridinium trifluoroacetate **S-MGB-207** (**17**)

6-[(*E*)-2-(4-Methoxyphenyl)ethenyl]-*N*-[1-methyl-5-({[1-methyl-5-({[2-(4-morpholinyl)ethyl]amino} carbonyl)- 1*H*-pyrrol-3-yl]amino}carbonyl)-1*H*-pyrrol-3-yl]nicotinamide **S-MGB-2 (5)**, (10 mg, 16.4 mmol) was dissolved in DMF (1 mL, anhydrous) to which we added *m*-CPBA (2.8 mg, 16.4 mmol). This was left to stir overnight after which it was purified directly by HPLC to obtain the desired product (10 mg, 82%). Purity after HPLC: >95%. M.p. >230 °C. n_max_ (KBr, cm^−1^): 3262, 3116, 3042, 2911, 1867, 1668, 1591, 1513, 1409, 1200, 1131. δ_H_ (DMSO-d_6_): 12.36 (1H, s), 10.47 (1H, s), 9.97 (1H, s), 9.06 (1H, s), 8.31 (1H, t, 5.8 Hz), 8.25 (1H, dd, *J =* 8.0, 2.5), 7.75 (2H, d, *J* = 16.0), 7.63–67 (3H, m), 7.34 (1H, d, *J =* 1.5), 7.2–7.27 (2H, m), 7.10 (1H, d, *J =* 1.5), 6.97–7.02 (3H, m), 3.95–3.98 (2H, m), 3.88 (3H, s), 3.81–3.85 (2H, m), 3.83 (3H, s), 3.80 (3H, s), 3.65–3.72 (4H, m), 3.28 (2H, m), 3.15 (2H, m). HRMS (FAB): found 628.2872; calculated for C_33_H_38_O_6_N_7_^+^: 628.2878.

##### {(E)-2-[4-({[1-methyl-5-({[1-methyl-5-({[2-(4-oxido-4-morpholinyl)ethyl]amino}carbonyl)-1H-pyrrol-3-yl]amino}carbonyl)-1H-pyrrol-3-yl]amino}carbonyl)phenyl]ethenyl}quinolinium trifluoroacetate **S-MGB-219** (**16**)

1-Methyl-*N*-[1-methyl-5-({[2-(4-morpholinyl)ethyl]amino}carbonyl)-1*H*-pyrrol-3-yl]-4-({4-[(*E*)-2-(3-quinolinyl)ethenyl]benzoyl}amino)-1*H*-pyrrole-2-carboxamide, **S-MGB-3 (6)**, (10 mg, 15.5 mmol) was dissolved in DMF (1 mL, anhydrous) to which we added *m*-CPBA (2.6 mg, 15.5 mmol). This was left to stir overnight after which it was purified directly by HPLC to obtain the desired product (8 mg, 68%). Purity after HPLC: >95%. M.p. >230 °C. n_max_ (KBr, cm^−1^): 3414, 3126, 2923, 2846, 1676, 1648, 1631, 1582, 1541, 1464, 1435, 1203, 1131, 1011. δ_H_ (DMSO-d_6_): 10.38 (1H, s), 9.98 (1H, s), 9.55 (1H, br), 8.41 (1H, d, *J =* 9.0), 8.22 (1H, t, *J* = 8.0), 7.87–8.03 (8H, m), 7.78 (1H, m), 7.56–7.66 (2H, m), 7.34 (1H, d, *J =* 1.7), 7.21 (1H, d, *J =* 1.7), 7.13 (1H, d, *J =* 1.7), 7.01 (1H, d, *J =* 1.7), 3.98–4.01 (2H, m), 3.88 (3H, s), 3.83 (3H, s), 3.62–3.69 (2H, m), 3.55–3.59 (4H, m), 3.27 (2H, m), 3.12 (2H, m). HRMS (FAB): found 648.29; calculated for C_36_H_38_O_5_N_7_^+^ (M + H): 648.29.

## Figures and Tables

**Table 1:** Biological evaluation of S-MGBs against *L. donovani*, *T. b. brucei* and *S. aureus*. Inhibition of hERG ion channel: compounds were assessed at 10 μM and % displacement of [3H]-astemizole is reported. Each assay was run in triplicate and 3 separate experiments were carried out (*n* = 3). * data previously reported [19]. ** Data previously reported [20].

S-MGB	*L. donovani* IC_50_ (μM)	*T. b. brucei* MIC (μM)	*S. aureus* MIC (μM)	hERG %
**1**	3.5 ± 0.1	0.78 *	50	65 **
**206**	2.4 ± 0.3	6.25	>100	44
**2**	5.7 ± 0.4	1.56 *	3.12	58 **
**207**	3.1 ± 1.0	12.5	>100	45
**3**	5.1 ± 0.7	<0.19 *	0.78	64 **
**219**	1.0 ± 0.7	3.12	>100	48

**Table 2:** DNA melting temperature increase against the oligomer d(5′-CGCATATATGCG-3′).

S-MGB-ID	ΔT_m_ (°C)
**1**	11
**206**	1
**2**	15
**207**	1
**3**	17
**219**	12

**Table 3:** HPLC Procedure for N-Oxides. The parameters below were used to purify N-Oxide S-MGBs using a flow rate of 6 mL/min.

Time (min)	% Water (with 0.1% TFA)	% MeCN (with 0.1% TFA)
0	70	30
25	50	50
30	70	30
35	70	30

## Data Availability

All data pertaining to this study have been reported herein. However, the authors are available to provide further information upon request.

## Supplementary Materials

The following supporting information can be downloaded at: https://www.mdpi.com/article/10.3390/ijms231911912/s1.
