# Supplementary material for: Selective Anti-Leishmanial Strathclyde Minor Groove Binders Using an N-Oxide Tail-Group Modification
Source: Int J Mol Sci. 2022 Oct 7;23(19):11912. doi: 10.3390/ijms231911912 (PMC9569768; doi:10.3390/ijms231911912)
Supplement: Supplementary file 1 [file ijms-23-11912-s001.zip › ijms-1934795-supplementary.pdf]

**Selective Anti-Leishmanial Strathclyde Minor Groove Binders Using an N-Oxide Tail Group Modification**

Marina C. Perieteanu[1], Leah M.C. McGee[1], Craig D. Shaw[2], Donna MacMillan[1], Abedawn I. Khalaf[1], Kirsten Gillingwater[3,4], Rebecca Beveridge[1], Katherine C. Carter[2], Colin J. Suckling[1], Fraser J. Scott[1,*]

[1]Department of Pure and Applied Chemistry, University of Strathclyde, Glasgow G1 1XL, United Kingdom
[2]Strathclyde Institute of Pharmacy & Biomedical Sciences, University of Strathclyde, Glasgow, G4 0RE, United Kingdom
[3]Parasite Chemotherapy, Department of Medical Parasitology and Infection Biology, Swiss Tropical and Public Health Institute, Basel 4051, Switzerland
[4]University of Basel, Basel 4001, Switzerland

# Supplementary Information

**Table S1.** Calculated and measured masses for each species observed in **Figure 4** for DNA sequence 5′-CGCATATATGCG-3′ **S-MGB-219.**

| Species | m/z value | Calculated mass of neutral species (Da) |
|---|---|---|
| **Single Stranded [SS]** | 3- : 1214.1<br>4- : 910.3 | (1214.1*3) + 3 = 3645.3<br>(910.3*4) + 4 = 3645.2 |
| **Double Stranded [DS]** | 4- : 1821.7<br>5- : 1457.2 | (1821.7*4) + 4 = 7290.8<br>(1457.2*5) + 5 = 7291.0 |
| ***Double Stranded + 2 x S-MGB-219 [DS+2M]*** | 4- : 2144.6<br>5- : 1715.5 | (2144.6*4) + 4 = 8582.4<br>(1715.5*5) +5 = 8582.5 |

**Table S2.** Calculated and measured masses for each species observed in **Figure 4** for DNA sequence 5′-CGCATATATGCG-3′ **S-MGB-BP-3.**

| Species | m/z value | Calculated mass of neutral species (Da) |
|---|---|---|

| | | |
|---|---|---|
| **Single Stranded [SS]** | 3- : 1214.1<br><br>4- : 910.3 | (1214.1*3) + 3 = 3645.3<br><br>(910.3*4) + 4 = 3645.2 |
| **Double Stranded [DS]** | 4- : 1821.7<br><br>5- : 1457.2 | (1821.7*4) + 4 = 7290.8<br><br>(1457.2*5) + 5 = 7291.0 |
| ***Double Stranded + 2 x S-MGB-BP-3 [DS+2M]*** | 4- : 2137.2<br><br>5- : 1709.6 | (2137.2) + 4 = 8552.8<br><br>(1709.6*5) +5 = 8553.0 |

For all species, the expected mass corresponds to the left-hand side of the m/z peaks.
